# Editorial: Food bioactive compounds in aging

**DOI:** 10.3389/fnut.2024.1395937

**Published:** 2024-03-22

**Authors:** Yoo Kim

**Affiliations:** Department of Nutritional Sciences, Oklahoma State University, Stillwater, OK, United States

**Keywords:** food bioactive compounds, aging, oxidative stress, life expectancy, lipid remodeling

The global demographic landscape is undergoing a profound transformation as the population ages at an unprecedented rate. With advancements in healthcare and technology, life expectancy has seen a notable rise, with the world average reaching 72.6 years as of 2019. However, this longevity comes with its own set of challenges, particularly concerning the quality of life in older age. In Europe, for instance, projections indicate a significant increase in the population aged 80 and over by 2080, highlighting the urgency for comprehensive strategies to address the needs of an aging society.

Aging is a multifaceted process characterized by a gradual decline in physiological function and resilience, leading to increased susceptibility to chronic diseases and diminished quality of life. While genetic factors play a role, lifestyle choices such as exercise and nutrition are pivotal in modulating the aging trajectory. The emerging field of “nutrigerontology” underscores the critical role of nutrition in promoting healthy aging and mitigating age-related ailments.

In this context, our Research Topic “*Food bioactive compounds in aging*” aims to shed light on various facets of aging and explore novel interventions that can enhance the wellbeing of older adults. We present a Research Topic of articles that delve into the intricate interplay between nutrition, lifestyle, and aging, offering valuable insights and potential avenues for intervention.

In our Research Topic, Kassis et al. “*Nutritional and lifestyle management of the aging journey: a narrative review*” provides a comprehensive overview of the physiological changes associated with aging and their implications for metabolic functions. By elucidating key biological targets, the review offers valuable perspectives on promoting healthy aging through strategic nutritional and lifestyle interventions. In a novel research endeavor, the original article titled “*Comparative assessment of Cucurbita moschata seed polypeptides toward the protection of human skin cells against oxidative stress-induced aging*” by Liu et al. explores the potential of *Cucurbita moschata* seed polypeptides in combating skin aging. The findings underscore the promising role of these polypeptides in mitigating oxidative stress-induced aging, offering new possibilities for skincare interventions. Furthermore, the research paper titled “*Partial replacement of high-fat diet with n-3 PUFAs enhanced beef tallow attenuates dyslipidemia and endoplasmic reticulum stress in tunicamycin-injected rats*” by Zheng et al. investigates the impact of dietary interventions on metabolic syndrome and endoplasmic reticulum stress. The study highlights the beneficial effects of incorporating *n-3* polyunsaturated fatty acids into the diet, emphasizing the potential of dietary modification in managing metabolic disorders associated with aging. Lastly, the randomized trial titled “*A randomized trial involving a multifunctional diet reveals systematic lipid remodeling and improvements in cardiometabolic risk factors in middle aged to aged adults*” by Arroyo et al. offers compelling evidence for the efficacy of a multifunctional diet in promoting metabolic health. Through systematic lipid remodeling, the multifunctional diet demonstrates promising outcomes in improving cardiometabolic risk factors independent of weight loss, underscoring the importance of dietary diversity in aging populations.

As we navigate the complexities of an aging population, it is imperative to adopt a holistic approach that encompasses nutrition, lifestyle modifications, and innovative interventions. By fostering interdisciplinary research and collaboration, we can unlock new strategies to enhance the health and wellbeing of older adults, paving the way for a healthier and more resilient aging population.

In conclusion, the articles presented in this Research Topic serve as a testament to the growing body of knowledge surrounding aging and underscore the importance of proactive measures in promoting healthy aging. As we confront the challenges posed by an aging population, let us continue to explore innovative solutions that empower individuals to age with grace and vitality.

## Author contributions

YK: Writing – original draft, Writing – review & editing.

